# ERRATUM: The impact of the final rinse on the cytoxicity of critical products submitted for processing

**DOI:** 10.1590/1980-220X-REEUSP-2024-ER12en

**Published:** 2024-09-30

In the article "The impact of the final rinse on the cytoxicity of critical products submitted for processing", with the DOI: https://doi.org/10.1590/S0080-623420150000700013, published by the journal: Revista da Escola de Enfermagem da USP, volume 49 (spe) of 2015, p. 86–91, on page 91:

**Now read:**

## Financial Support

This study was financed in part by the Coordenação de Aperfeiçoamento de Pessoal de Nível Superior – Brasil (CAPES) – Finance Code 001.

