# Supplementary material for: ERRATUM: The impact of the final rinse on the cytoxicity of critical products submitted for processing
Source: Rev Esc Enferm USP. 2024 Sep 30;58:e2024ER12. doi: 10.1590/1980-220X-REEUSP-2024-ER12en (PMC11524268; doi:10.1590/1980-220X-REEUSP-2024-ER12en)
Supplement: Translation

# Translation: ERRATA: O impacto do último enxágue na citotoxicidade de produtos críticos passíveis de processamento

No artigo “O impacto do último enxágue na citotoxicidade de produtos críticos passíveis de processamento”, com número de: DOI: https://doi.org/10.1590/S0080-623420150000700013, publicado na Revista da Escola de Enfermagem da USP, volume 49 (Esp) de 2015, p. 86–91, na página 91:

**Leia-se:**

## Apoio financeiro

O presente trabalho foi realizado com apoio da Coordenação e Aperfeiçoamento de Pessoal de Nível Superior – Brasil (CAPES) – Código de Financiamento 001.
